# Risk factors for bone metastasis in patients with primary lung cancer: A systematic review

**DOI:** 10.1097/MD.0000000000014084

**Published:** 2019-01-18

**Authors:** Yujie Niu, Yiting Lin, Hailin Pang, Weiwei Shen, Lili Liu, Helong Zhang

**Affiliations:** a Department of Oncology, Tangdu Hospital, Fourth Military Medical University, Xi’an, Shaanxi; b Department of Oncology and Hematology, Ningxia People's Hospital; c Department of Oncology and Hematology, The First Affiliated Hospital of Northwest University to Nationalities, Yinchuan, Ningxia; d Cancer Institute, the Fourth Military Medical University, Xi’an, Shaanxi, China.

**Keywords:** bone metastasis, lung neoplasms, risk factors, skeletal-related events, systematic review

## Abstract

**Background:**

Bone metastases (BM) are prevalent among lung cancer (LC) patients. Although some studies revealed associated factors for BM, each of these papers focused on a few factors. Few studies have identified the potential risk factors through a systematic review.

**Methods:**

We searched through PubMed, MEDLINE, Web of Science, EMBASE, Cochrane Library and Cochrane Central Register

of Controlled Trials for literature from January 1990 to November 2017. The types of literature included case–control studies, cohort studies, randomized controlled trials and systematic reviews.

**Results:**

From included 12 studies, we identified that lower blood calcium, T4 stage, N3 stage, P-stage III, nonsquamous, bone sialoprotein expression, elevated carcino-embryonic antigen levels were risk factors for bone metastasis in lung cancer patients.

**Conclusion:**

We identified that T4 stage, N3 stage, and positive bone sialoprotein expression associated with an increased risk of bone metastasis. Further studies are needed to assess these relationships and to establish the risk prediction model of bone metastasis.

## Abstract 2

Supplemental Digital Content is available in the text

## 1 Introduction

Bone metastases (BM) are prevalent among lung cancer (LC) patients.^[1]^ Around 30% to 40% of LC patients develop BM in the disease course.^[2]^ BM would cause severe complications, like pathological fractures, spinal cord compression, hypocalcemia and other skeletal-related events (SREs).^[3]^ Each of them would bring about a rising cost of healthcare and the impaired quality of life.^[4,5]^ Skeletal metastases account for approximately 350,000 deaths in the United States every year,^[6]^ and nearly 3 times this number if patients in the European countries and Japan are also included. Early treatments are effective to lower the incidence of complications and medical expenses.^[5]^ Applications of bisphosphonates and denosumab might relieve suffering and save money for every LC patients.^[7–9]^ It would be a turning point for every patient's wellbeing if we can find out risk factors for BM/SREs. We have many high-tech types of equipment that can find out bone lesions of BM/SREs, but no one could identify the latent hazard. Therefore, it is very imperative for us to identify risk factors of BM/SREs before things get worse than before. Thankfully, many researchers have done plenty of work on this topic.

There have been some studies of risk factors for BM in lung cancer. In 1999, Kobayashi et al^[10]^ identified that the aminoterminal propeptide of Type I collagen (PINP) and carboxyterminal telopeptide of Type I collagen (ICTP) correlated with BM and survival time. They appeared to be of great value for the prediction of BM. In 2005, Brown et al^[11]^ found that bone biomarker levels were an indicator of SREs, disease progression and death in patients with BM secondary to nonsmall cell lung cancer (NSCLC). In the next year, Coleman et al^[12]^ published an article that the bone resorption marker NTX provided predictive information in BM patients. They found that high NTX levels (≥100 nmol/mmol creatinine) were related to high risk of SREs and disease progression compared with low NTX levels (<50 nmol/mmol creatinine).

However, these studies all focused on a few factors. Previous studies have shown that expression of some biochemical compounds (e.g., bone sialoprotein, osteopontin, and N-telopeptide of type I collagen (NTX), serum cross-linked carboxyterminal telopeptide of type I collagen (ICTP) and the aminoterminal propeptide of type I collagen (PINP)) strongly associated with development and progression of BM in lung cancer patients.^[10–16]^ It is not enough to involve these biomarkers in predicting the incidence of BM/SREs. We need more evidence to recognize factors to apply them in identifying the high-risk population. This systematic review intended to help clinicians generate a basic conceptual structure to better understand the relationship between potential risk factors and BM/SREs.

## 2 Materials and methods

### 2.1 Electronic search

We applied PubMed, MEDLINE, Web of Science, EMBASE, the Cochrane Library (Cochrane Database of Systematic Reviews) and the Cochrane Central Register of Controlled Trials (CENTRAL) (from January 1990 to November 2017) to search the relevant literature without any language restrictions. We used predefined keywords to run searches: “primary pulmonary neoplasm,” “risk factors,” and “bone metastases.” We described search strategy for PubMed in detail in Supplementary File 1. Primary and secondary outcomes should be BM and SREs. We summarized the effect estimates of risk factors and used random-effect models to pool the estimates if the outcomes and characteristics in studies were comparable. The quality of the study was assessed using the Newcastle–Ottawa Scale and the Cochrane Collaboration tool.^[17]^

### 2.2 Selection criteria and data collection

We included case–control, cohort studies, randomized controlled trials (RCTs) and systematic reviews in adults and elderly patients with primary lung cancer. Descriptions of risk factors are adequate. The primary and secondary outcomes are BM and SREs separately. BM is defined as one or more radiographically confirmed bone metastases. Diagnostic methods include plain radiography, myelography, MRI, CT, radionuclide bone scanning (scintigraphy with technetium-99m-labeled diphosphonates), single-photon emission CT and positron emission tomography.^[18]^ SREs include the first SRE, time-to-the first SRE, all SREs, SRE-free survival, skeletal progression and related death (our protocol described SREs in detail^[17]^).

We retrieved information for eligible studies (the PRISMA guidelines, www.prisma-statement.org)^[19]^ using a predefined procedure and collection form.^[17]^ The heterogeneity of study design and outcomes did not fit for a meta-analysis, so we undertook a systematic narrative review to synthesize potential risk factors of BM/SREs. Experimental procedures were approved by the Institutional Review Board of the Fourth Military Medical University.

### 2.3 Study characteristics

**Figure 1:**
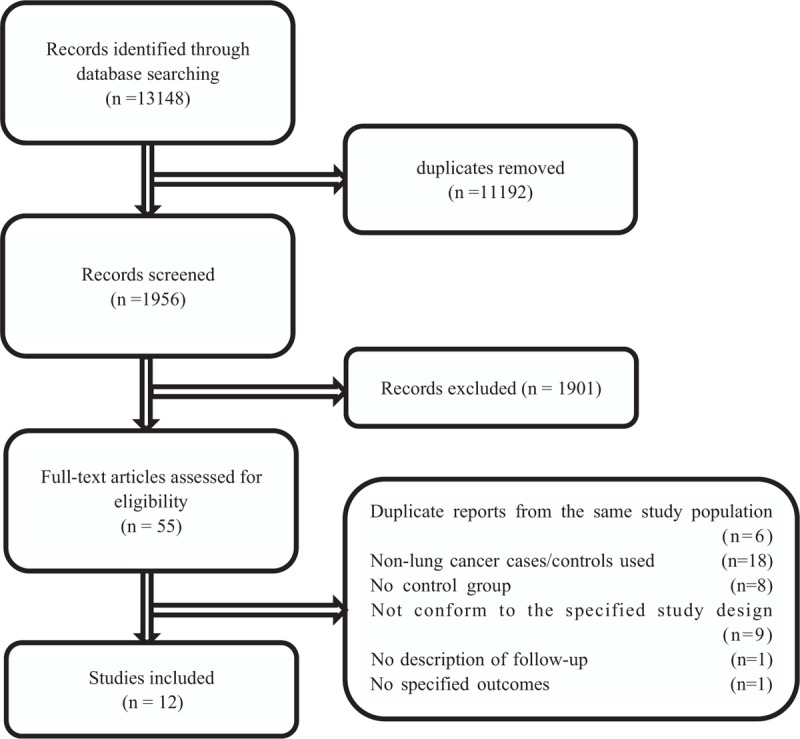
Flow chart of the selection of studies.

Through database searching, we identified 13,148 references. We used Endnote (Microsoft, Redmond, WA) to remove 11,192 duplicates. Then 2 review authors (W-WS, Y-TW) separately examined 1956 publications. After exclusion of inconsistent titles/abstracts, getting full-text of publications, uniting different articles of the same study together, and analyzing full-text based on eligibility criteria, we listed 12 final selected publications. After assessment of eligibility, 6 records were duplicate reports from the same study population; 18 records were nonlung cancer case/control groups; 8 references had no control group; 9 studies did not conform to the specified study design. The flowchart (Fig. 1) presented the specific selection processes.^[12–14,20–28]^

Table 1 shows the characteristics of selected studies. Among the 12 eligible studies, 2 of them are multicentre studies, which were carried out by cooperative groups. We included 4179 patients and analyzed 3580 patients of them. Among 6 publications which displayed the tumor node metastasis (TNM) staging of patients with lung cancer, a median of 19.7% (range, 12.2%–66.3%) of patients had Stage I/II, and a median of 90.15% of patients (range, 26.7%–100%) had Stage III/IV disease. Though Coleman et al^[12]^ shared the same database of a randomized controlled trial^[29]^ with Hirsh et al,^[22]^ their purposes and populations were varied. These studies had different limits of NTX levels among patients with placebo on zoledronic acid.^[12,22,29]^

**Table 1:**
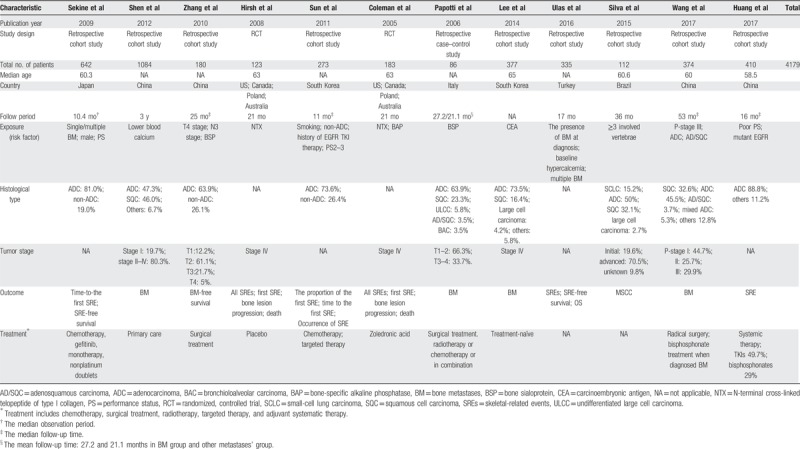
Characteristics of studies related to bone metastases/skeletal-related events in lung cancer patients.

### 2.4 Risk of bias assessment

Two investigators (SW, XB) separately assessed the risk of bias (ROBs) using the Newcastle-Ottawa scale^[30]^ and the Cochrane Collaboration tool^[31]^ to value observational studies and RCTs, separately. We contacted authors of publications with open-ended questionnaires for additional information if some data were needed.

The publication of Lee et al^[28]^ got the least score using the Newcastle-Ottawa Scale. Although both articles of Coleman et al^[12]^ and Hirsh et al^[22]^ came from one original study,^[29]^ we treated them as 2 studies because they had diverse purposes and populations. However, bias from selective reporting of outcomes was likely to occur in the study of Hirsh et al. Table 2 presents ROB ratings and scores for included observational studies, which indicated the need for high-quality articles.

**Table 2:**
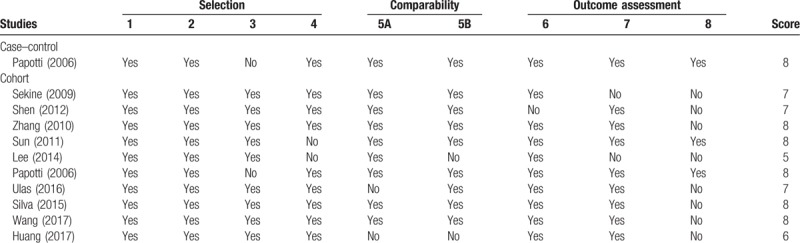
Risk of bias assessment of observational studies.

## 3 Results

### 3.1 Risk factors for bone metastasis

#### 3.1.1 Lower blood calcium

One eligible article^[23]^ noted an increased risk of BM among resected NSCLC patients who were accompanied with lower blood calcium (<2.2 μM) (vs 2.2 μM ≤ blood calcium ≤ 2.6 μM) (unadjusted relative risk (RR): 2.039, 95% CI: 1.395–2.981; *P* < .01).

#### 3.1.2 T4 stage

One study^[13]^ reported an increased risk of bone metastases with T4 stage (vs T1, 2, and 3) among completely resected primary NSCLC cases (hazard ratio (HR): 1.618, 95% CI: 1.064–2.460; *P* = .024). In an analysis of BM-free survival, it demonstrated that the T4 stage was an independent factor for bone metastasis (Table 3).

**Table 3:**
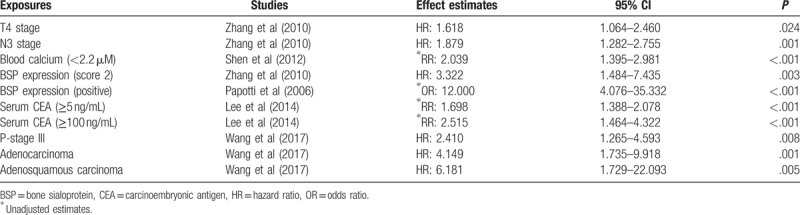
Association between exposure to potential risk factors and risk of bone metastasis.

#### 3.1.3 N3 stage

Zhang et al^[13]^ demonstrated an effect of the N3 stage (vs N0, 1, and 2) among patients of completely resected NSCLC (HR: 1.879, 95% CI: 1.282–2.755; *P* = .001) (Table 3).

#### 3.1.4 P-stage III

From the study of 374 NSCLC patients, Wang et al^[24]^ evaluated that P-stage III was a high-risk factor influencing bone metastasis. Univariate analysis suggested that P-stage III (*P* = .007) was an independent factor for BM comparing P-stage I + II. A multivariate analysis found that patients with P-stage III had a higher risk for bone metastasis (HR: 2.410; 95% CI: 1.265–4.593; *P* = .008) than P-stage I + II. There were no significant differences between patients with P-stage I disease and patients with P-stage II disease (HR: 1.089; 95% CI: 0.482–2.461; *P* = .838). All above suggested P-stage III was related to high risk of BM in NSCLC patients.

#### 3.1.5 Nonsquamous

One study^[24]^ explored whether pathological types affected BM in NSCLC patients or not. Compared with squamous cell carcinoma, the HRs for adenocarcinoma, adenosquamous carcinoma, mixed adenocarcinoma, and other pathological types (e.g., large cell carcinoma and atypical carcinoid) were 4.149 (*P* = .001), 6.181 (*P* = .005), 2.754 (*P* = .273), and 0.951 (*P* = .951), respectively. Adenocarcinoma patients had the highest risk of bone metastasis.

#### 3.1.6 Positive BSP expression

Two studies identified this variable. Papotti et al^[14]^ found that positive bone sialoprotein (BSP) expression (score 2 vs score 1) strongly correlated with the development of BM (unadjusted odds ratio (OR): 12.000, 95% CI: 4.076–35.332; *P* < .001). In another retrospective cohort study,^[13]^ Zhang et al confirmed that positive BSP expression (vs negative) was related to bone metastases in NSCLC (HR: 3.322, 95% CI: 1.484–7.435; *P* = .003) (Table 3).

#### 3.1.7 Elevated CEA levels

One study^[28]^ showed that elevated serum carcino-embryonic antigen (CEA) levels (≥5 ng/mL vs normal CEA levels) correlated with increased BM risk (unadjusted RR: 1.698, 95% CI: 1.388–2.078; *P* < .001). Moreover, patients with very high serum CEA levels (≥100 ng/mL) were at higher risk for BM than abnormal serum CEA levels (<100 ng/mL) (unadjusted RR: 2.515, 95% CI: 1.464–4.322; *P* < .001) (Table 3).

### 3.2 Risk factors of SREs

#### 3.2.1 Ever-smoking

One research^[21]^ reported a higher SREs risk in ever-smokers (OR: 2.80, 95% CI: 1.32–6.00; *P* = .007). After excluding patients with prophylactic bisphosphonate treatment before first SRE, they still found an increased risk of SREs in ever-smokers (OR: 2.09, 95% CI: 1.19–3.65; *P* = .010). Using the analysis of time-to-the first SRE, they found that the median time of ever-smoker was statistically significantly reduced (5.2 months vs 11.6 months; HR:1.75, 95% CI:1.05–2.92; *P* = .030). In the multiple-event analysis, it revealed a higher risk of SREs in ever-smokers (HR: 1.601, 95% CI: 1.034–2.479; *P* = .035) (Table 4).

**Table 4:**
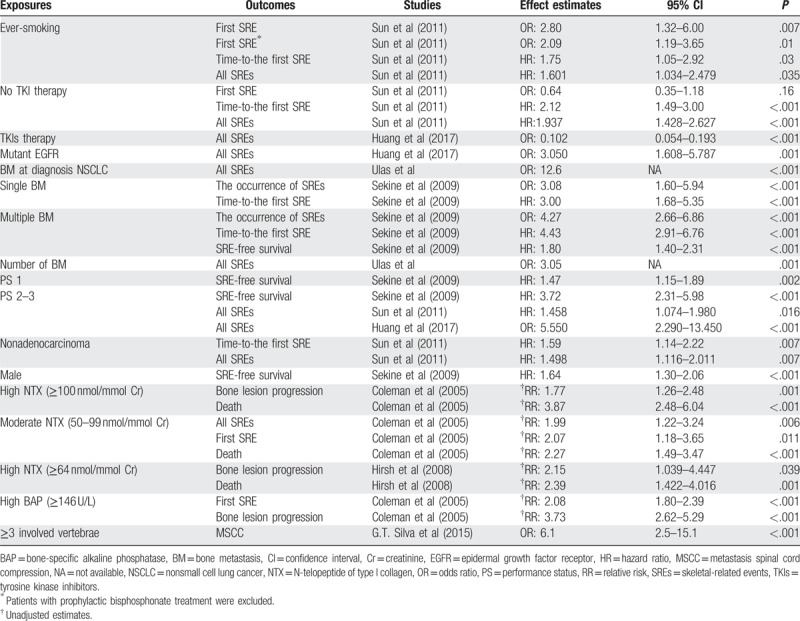
Association between exposure to potential risk factors and risk of skeletal-related events.

#### 3.2.2 No history of EGFR-TKIs therapy

A cohort study^[21]^ reported a higher risk of all SREs in patients with no history of treatment with epidermal growth factor receptor (EGFR) tyrosine kinase inhibitors (TKIs)(HR: 1.937, 95% CI: 1.428–2.627; *P* < .001). It displayed that median time-to-the first SRE of these patients were significantly reduced (3.3 months vs 11.8 months; HR: 2.12, 95% CI: 1.49–3.00; *P* < .001). However, in logistic regression analysis, it reported a null effect of patients with no history of treatment with EGFR TKIs (OR: 0.64, 95% CI: 0.35–1.18; *P* = .160) (Table 4).

#### 3.2.3 History of radiotherapy to the bone before chemotherapy

For the rate of occurrence of SREs, Sekine et al^[20]^ revealed a null effect of History of Radiotherapy to the Bone before Chemotherapy (RTB) (OR: 1.43, 95% CI: 0.69–2.97; *P* = .336). For time-to-the first SRE, they still revealed a null result of RTB versus no RTB (HR: 1.39, 95% CI: 0.77–2.49; *P* = .275). Additionally, the result of SRE-free survival was similar (HR: 1.10, 95% CI: 0.71–1.71; *P* = .670).

#### 3.2.4 Multiple BM

Both Sekine et al and Ulas et al reported the number of BM associated with SREs.

Sekine et al^[20]^ found the number of BM at the time of the initial diagnosis influenced the occurrence of SREs. They reported that single BM (vs none BM) (OR: 3.08, 95% CI: 1.60–5.94; *P* < .001) and multiple BM (vs none BM) (OR: 4.27, 95% CI: 2.66–6.86; *P* < .001) associated with an increased risk of SREs. For time-to-the first SRE, it showed similar effects of single BM (HR: 3.00, 95% CI: 1.68–5.35; *P* < .001) and multiple BM (HR: 4.43, 95% CI: 2.91–6.76; *P* < .001). But for SRE-free survival, only the result of multiple BM was statistically significant (HR: 1.80, 95% CI: 1.40–2.31; *P* < .001) (Table 4). Then, in 2016, Ulas et al identified the number of BM as a significant factor in predicting SREs (OR: 3.05), such as the need for radiotherapy and malignant hypercalcemia.

#### 3.2.5 PS

Two studies reported performance status (PS). For time-to-the first SRE, Sekine et al^[20]^ revealed null results of PS of 1 versus 0 (HR: 1.15, 95% CI: 0.76–1.74; *P* = .510) and PS of 2/3 (vs 0)(HR: 2.21, 95% CI: 0.97–5.03; *P* = .059). Sun et al^[21]^ also demonstrated null results of Eastern Cooperative Oncology Group (ECOG) PS of 0/1/2/3 (effect estimates not available in the original article).

However, for the SRE-free survival,^[20]^ it displayed an increased SRE’ risk of PS of 1 (HR: 1.47, 95% CI: 1.15–1.89; *P* = .002). Using multivariate analysis^[20]^ showed an increased risk of PS of 2/3 versus 0 (HR: 3.72, 95% CI: 2.31–5.98; *P* < .001). Additionally, Sun et al^[21]^ showed a similar trend of ECOG PS of 2/3 (vs ECOG 0/1) (HR: 1.458, 95% CI: 1.074–1.980; *P* = .016) (Table 4).

#### 3.2.6 Nonadenocarcinoma

Sun et al^[21]^ found that nonadenocarcinoma was significantly associated with higher risk of patients with time-to-the first SRE (HR:1.59, 95% CI: 1.14–2.22; *P* = .007). Besides, they observed an increased risk of SREs in patients with nonadenocarcinoma versus adenocarcinoma (HR: 1.498, 95% CI: 1.116–2.011; *P* = .007). However, it displayed an inconsistent effect for the proportion of first SRE, which showed a null result of nonadenocarcinoma (OR: 1.55, 95% CI: 0.83–2.87; *P* = .170) (Table 4).

#### 3.2.7 Gender

Sekine et al^[20]^ revealed a null result of male versus female (HR: 1.44, 95% CI: 0.98–2.11; *P* = .063). However, they found that male patients had an increased risk for SRE-free survival (HR: 1.64, 95% CI: 1.30–2.06; *P* < .001). Like the previous result, Sun et al^[21]^ reported a null effect of a male for the proportion of the first SRE (OR: 0.68, 95% CI: 0.32–1.45; *P* = .28). It^[21]^ revealed that there is no statistically significant increase in the risk of SREs in female patients (HR: 1.382, 95% CI: 0.879–2.170; *P* = .161) (Table 4).

#### 3.2.8 Elevated NTX levels

Two studies^[12,22]^ presented the effect of urinary NTX levels on NSCLC. Hirsh et al^[22]^ reported a null result of elevated NTX levels (≥64 nmol/mmol Cr) in placebo treated patients (vs normal NTX levels) (unadjusted RR: 1.64, 95% CI: 0.964–2.790, *P* = .068; unadjusted RR: 1.49, 95% CI: 0.782–2.838, *P* = .225, respectively). But it showed strong associations of elevated NTX levels with bone lesion progression/death (unadjusted RR: 2.15, 95% CI: 1.039–4.447, *P* = .039; unadjusted RR: 2.39, 95% CI: 1.422–4.016, *P* = .001, separately).

Another study^[12]^ analyzed this relationship in zoledronic acid treated patients. It investigated increased bone lesion progression/death risks of high NTX (≥100 nmol/mmol Cr) vs low NTX (unadjusted RR: 1.77, 95% CI: 1.26–2.48, *P* = .001; unadjusted RR: 3.87, 95% CI: 2.48–6.04, *P* < .001, respectively). But for All SREs/First SRE, it suggested null results of high NTX levels (unadjusted RR: 1.89, 95% CI: 0.86–4.15, *P* = .111; unadjusted RR: 1.56, 95% CI: 0.67–3.64, *P* = .306, separately).

Notwithstanding it reported a null effect for bone lesion progression (unadjusted RR: 1.40, 95% CI: 0.74–2.65; *P* = .294), generally, moderate NTX levels (50–99 nmol/mmol Cr) were correlated with a higher risk for SREs. Furthermore, it suggested an increased All SREs’ risk for patients with moderate NTX levels (unadjusted RR: 1.99, 95% CI: 1.22–3.24; *P* = .006), which also associated with a higher risk of first SRE for the stage IV patients (unadjusted RR: 2.07, 95% CI: 1.18–3.65; *P* = .011). It also showed a 2.27-fold increased risk of death with moderate NTX levels (unadjusted RR: 2.27, 95% CI: 1.49–3.49; *P* < .001).

#### 3.2.9 Elevated BAP levels

Coleman et al^[12]^ reported the effect of bone-specific alkaline phosphatase (BAP) on the risk of SREs among patients treated with zoledronic acid. Generally, elevated BAP levels (≥146 U/L) correlated with a higher risk for SREs irrespective of outcomes. However, the correlation was weak for All SREs (unadjusted RR: 1.29, 95% CI: 0.89–1.88; *P* = .180).

#### 3.2.10 Presence of bone metastasis at diagnosis

Ulas et al^[26]^ followed up 835 NSCLC patients and found the presence of bone metastasis at diagnosis was a predictive factor of SREs (OR: 12.6). The most common SREs were the need for radiotherapy (43.2%) and malignant hypercalcemia (17.6%). The median time to first SRE was 3.5 months at the median follow-up of 17 months.

#### 3.2.11 Three or more metastatic vertebrae

An article^[27]^ revealed lung cancer patients with 3 or more metastatic vertebrae had a great risk of developing metastatic spinal cord compression (MSCC) than those who have up to 2 involved vertebrae (OR:6.1, 95% CI:2.5–15.1, *P* < .001).

## 4 Discussion

Bone metastasis and SREs are frequent and burdensome among lung cancer patients. Although we can apply multiple approaches to diagnose BM/SREs, clinicians need comprehensive and systematic information to predict the risk factors of BM/SREs and to decide the suitable strategies for preventing and treating disease. Therefore, it is imperative to identify the potential risk factors of BM/SREs from previous studies. Recently, new predictors provided directions to prevent BM/SREs; we need an accurate prediction model to estimate risk.

In an exploratory cohort analysis published in 2005, Coleman et al^[12]^ suggested that elevated NTX or BAP levels positively correlated with SREs. In 2006, another retrospective case–control study reported that BSP protein expression was positively associated with higher risk of BM progression and may be a useful predictor in identifying a high-risk population in the primary resected NSCLC.^[14]^ In a retrospective cohort study published in 2009, Sekine et al^[20]^ found that multiple BM was strongly correlated with increased risk of SREs in advanced NSCLC. They also suggested that male and poor PS were additional predictors for SRE-free survival. In 2010, Zhang et al^[13]^ confirmed that positive BSP expression in the primary resected Chinese NSCLC positively associated with a higher risk of BM. It was coincident with Papotti et al,^[14]^ who concluded that there was a positive correlation between BSP expression and BM. Besides, this cohort study also revealed that the T4 stage and N3 stage were independent risk factors for BM. In a retrospective cohort study published in 2011, Sun et al^[21]^ found that patients ever-smoking had a significantly higher risk of SREs than never-smokers. Another cohort study from China indicated that decreased blood calcium levels at initial care associated with an increased risk of BM versus normal levels in 2012.^[23]^ Besides, in 2014, Lee et al^[28]^ showed that increased serum CEA levels could be a predictor of increased bone metastatic potential in stage IV lung cancers.

Thus, despite the individual researches reporting developments of predicting BM/SREs, our review set out to provide a pooled analysis of the expected improvements. We ran this systematic review and evaluated articles published from 1990 to 2014. There seemed to be progressive developments in risk factors of BM/SREs in lung cancers. We found that T/N staging and positive BSP expression positively associated with the occurrence of BM. Moreover, results showed that ever-smoking and multiple BM correlated with the proportion of SREs.

The literature supports these associations. Our results are consistent with the large-scale correlative literature of bone turnovers in patients with solid tumors (including NSCLC) published in 2005.^[11]^ Brown et al^[11]^ found that recent bone turnover assessments (e.g., NTX, BAP) were better indicators for SREs than baseline bone marker levels. Then in 2013, Sutcliffe et al^[18]^ reported that there was an increased likelihood of SREs with ever-smoking, lack of history of therapy with EGFR TKIs, poor ECOG status, and nonadenocarcinoma. Their review found that the greater the number of BM, the higher was the risk of SREs. In an exploratory analysis published in 2013, Lipton et al^[32]^ showed that biomarkers of bone metabolism could provide insight into ongoing rates of bone destruction among patients with malignant skeletal diseases.^[33]^ However, from their previous studies, inconsistent results gave us confused interpretations about baseline NTX levels.^[34,35]^ In this retrospective analysis, among patients with BM from prostate cancer, breast cancer, NSCLC or other solid tumors who received zoledronic acid treatment, Lipton et al^[32]^ suggested that NTX elevations not precede SREs. By contrast, our study focuses on lung cancers, despite mixed solid tumors in previous studies.

We admit that our study has some limitations. Originally, in our protocol, we intended to conduct a systematic review.^[17]^ Only when several studies have the same risk factor, we would perform a meta-analysis.^[17]^ Because included studies have different risk factors, we cannot synthesize their results. Second, the majority of included studies were retrospective studies. These data are probably lack of accuracy because patients cannot always remember when and how frequently they were exposed to risk factors. Third, it is possible that we did not include studies which could affect the result and conclusion in the current analysis. Another limitation of the observational studies was that it was not possible to control all potential confounding covariates. Because most of the included studies were observational studies, this bias was inevitable but did not have a major effect on the results of the analysis.

Despite these limitations, the evidence from this review may help establish risk prediction models (RPMs) for BM/SREs in lung cancers and apply these predictors to identify the high-risk population. Our finding provides comprehensive and systematic information to help oncologists identify patients who might obtain a benefit from systematic therapy and to help clinicians to prevent BM and SREs in future works.

In conclusion, this review has made several conclusions about clinical problems. Lung cancer patients with T4 stage, N3 stage, and positive BSP expression may experience a higher risk of BM. Furthermore, our data showed that ever-smoking and multiple BMs significantly associated with an increased risk of SREs in lung cancer patients with BM.

## Author contributions

**Conceptualization:** Helong Zhang.

**Data curation:** Yujie Niu.

**Formal analysis:** Yujie Niu.

**Funding acquisition:** Helong Zhang.

**Investigation:** Weiwei Shen.

**Methodology:** Yujie Niu, Lili Liu, Helong Zhang.

**Supervision:** Yiting Lin.

**Validation:** Yiting Lin.

**Writing – original draft:** Yujie Niu.

**Writing – review & editing:** Yiting Lin, Hailin Pang.

## Supplementary Material

Supplemental Digital Content
