# Supplementary material for: Risk factors for bone metastasis in patients with primary lung cancer: A systematic review
Source: Medicine (Baltimore). 2019 Jan 18;98(3):e14084. doi: 10.1097/MD.0000000000014084 (PMC6370015; doi:10.1097/MD.0000000000014084)
Supplement: Supplemental Digital Content [file medi-98-e14084-s001.docx]

# PubMed Search Strategy

#1 Small Cell Lung Carcinoma [MeSH]

#2 Carcinoma, Non-Small-Cell Lung [MeSH]

#3 Lung Neoplasms [MeSH]

#4 Primary pulmonary neoplasm [TW]

#5 Primary lung cancer [TW]

#6 Lung cancer

#7 or/1-6

#8 Bone and Bones [MeSH]

#9 Neoplasm Metastasis [MeSH]

#10 Bone metastases [TW]

#11 Bone metastasis [TW]

#12 Bone metastases

#13 Bone metastasis

#14 or/8-13

#15 Humans [MeSH]

#16 Humans [TW]

#17 or/15-16

#18 Risk Factors [MeSH]

#19 Risk Factors [TW]

#20 Predictive value [TW]

#21 Predictive factors [TW]

#22 Predictive model [TW]

#23 Predictive biomarkers [TW]

#24 Predictive biomarker [TW]

#25 Biomarkers

#26 Early detection

#27 Risk factors

#28 Factors

#29 or/18-28

#30 7 and 14 and 17 and 29

Limit #30 to yr=“1990-current”
